# Copy number variations in candidate genomic regions confirm genetic heterogeneity and parental bias in Hirschsprung disease

**DOI:** 10.1186/s13023-019-1205-3

**Published:** 2019-11-25

**Authors:** Francesca Lantieri, Stefania Gimelli, Chiara Viaggi, Elissavet Stathaki, Michela Malacarne, Giuseppe Santamaria, Alice Grossi, Manuela Mosconi, Frédérique Sloan-Béna, Alessio Pini Prato, Domenico Coviello, Isabella Ceccherini

**Affiliations:** 1 Dipartimento di Scienze della Salute, sezione di Biostatistica, Universita’ degli Studi di Genova, 16132 Genoa, Italy; 2 Department of Medical Genetic and Laboratories, University Hospitals of Geneva, Geneva, Switzerland; 3 S.C. Laboratorio Genetica Umana, Ospedali Galliera, Genoa, Italy; 4 U.O.C. Genetica Medica, IRCCS, Istituto Giannina Gaslini, 16148 Genoa, Italy; 5 UOC Chirurgia Pediatrica, Istituto Giannina Gaslini, 16148 Genoa, Italy; 6 Present address: Children Hospital, AON SS Antonio e Biagio e Cesare Arrigo, Alessandria, Italy; 7 Present address: U.O.C. Laboratorio di Genetica Umana, IRCCS Istituto Giannina Gaslini, Genoa, 16148 Italy

**Keywords:** Hirschsprung disease, Copy number variations, Comparative genomic hybridization, Custom array, Candidate genes and regions

## Abstract

**Background:**

Hirschsprung Disease (HSCR) is a congenital defect of the intestinal innervations characterized by complex inheritance. Many susceptibility genes including *RET*, the major HSCR gene, and several linked regions and associated loci have been shown to contribute to disease pathogenesis. Nonetheless, a proportion of patients still remains unexplained. Copy Number Variations (CNVs) have already been involved in HSCR, and for this reason we performed Comparative Genomic Hybridization (CGH), using a custom array with high density probes.

**Results:**

A total of 20 HSCR candidate regions/genes was tested in 55 sporadic patients and four patients with already known chromosomal aberrations. Among 83 calls, 12 variants were experimentally validated, three of which involving the HSCR crucial genes SEMA3A/3D, NRG1, and PHOX2B. Conversely *RET* involvement in HSCR does not seem to rely on the presence of CNVs while, interestingly, several gains and losses did co-occur with another *RET* defect, thus confirming that more than one predisposing event is necessary for HSCR to develop. New loci were also shown to be involved, such as ALDH1A2, already found to play a major role in the enteric nervous system. Finally, all the inherited CNVs were of maternal origin.

**Conclusions:**

Our results confirm a wide genetic heterogeneity in HSCR occurrence and support a role of candidate genes in expression regulation and cell signaling, thus contributing to depict further the molecular complexity of the genomic regions involved in the Enteric Nervous System development. The observed maternal transmission bias for HSCR associated CNVs supports the hypothesis that in females these variants might be more tolerated, requiring additional alterations to develop HSCR disease.

## Background

Hirschsprung Disease (HSCR) (OMIM# 142623) is a congenital intestinal aganglionosis caused by a premature arrest of the cranio-caudal migration of neural crest cells during embryogenesis, showing an incidence of around 1/5000 live births [1]. The phenotype is highly variable, with severity classified according to the length of the affected gastrointestinal tract as short-segment HSCR (aganglionosis does not extend beyond the upper sigmoid, S-HSCR: 80% of cases) and long-segment HSCR (L-HSCR: 20% of cases). About 70% of cases presents as isolated, while 30% shows additional anomalies, including chromosomal aberrations [1]. HSCR is characterized by increased sibling recurrence risk varying by gender, length of aganglionosis and familial occurrence (up to 80% of cases are sporadic). These observations, together with a distorted sex ratio (M:F **=** 4:1), make HSCR a model for complex genetic disease. Genetic heterogeneity in HSCR is demonstrated by involvement of several genes and loci [2–4]. The major gene involved in isolated HSCR is the *RET* proto-oncogene [1], located on 10q11.2 and linked to HSCR in 90% of the familial forms [3]. In addition, HSCR has been associated with several *RET* polymorphisms, most of which part of a common risk haplotype encompassing the RET gene from the promoter to exon 2 [5–8]. An association with *NRG1* (OMIM# 142445) and *SEMA3A* (OMIM# 603961) */ SEMA3D* (OMIM# 609907) has also been described [9–11]. However, a proportion of HSCR patients still remains unexplained as only 50% of familial and 7–35% of sporadic HSCR cases can be explained by *RET* coding variants [1]. Deletions in genes already known to be involved in HSCR might account for these latter cases. Indeed, the *RET* gene was discovered following observation of de novo interstitial deletions of 10q11.2 [12, 13] and about 12% of HSCR patients have structural abnormalities [1].

Copy number variations (CNVs), already proven to be genetic risk factors in disease pathogenesis [14, 15], might thus account for part of the missing heritability in HSCR. Jiang et al. (2011) performed a custom-designed array CGH to examine 67 candidate HSCR genes in 18 HSCR patients, identifying seven CNVs at three loci, all likely hosting regulatory genes in syndromic HSCR patients [16]. On the other hand, Tang et al. (2012) assessed the CNV contribution to HSCR from genome-wide SNP data finding a greater burden for rare CNVs in HSCR cases over controls and larger CNVs in syndromic HSCR than in isolated cases. Only six CNVs overlapped with known HSCR loci, none involving the *RET* gene [17]. Another study investigated 123 HSCR patients and 432 unaffected subjects, with Illumina’s HumanOmni1-Quad BeadChip, finding 16 CNV regions associated with HSCR [18]. Finally, very recently, Tilghman et al. (2019) have dissected, through both karyotyping and exome sequencing, the differential contribution to HSCR development of three different molecular classes of risk alleles, namely rare coding variants, common non coding variants and large CNVs and chromosomal anomalies. In this latter class, at least 9 loci have been reported, whose aberrations showed a very high odd ratio (63.07) and are involved in 11.4% of the patients [19].

To further explore genetic heterogeneity in HSCR, we have performed CGH, using a custom array with high density probes and focusing on a total of 20 candidate regions/genes already known to be involved in HSCR, on a selected panel of 55 sporadic HSCR previously genotyped at the *RET* locus [6] and four HSCR patients with already known chromosomal aberrations.

## Results

### HSCR patients and regions analyzed

A total of 55 Italian sporadic HSCR patients fully genotyped at the *RET* locus [6] were retrospectively included in the study. To investigate the possible presence of interstitial deletions of the *RET* region, we selected 52 cases homozygous for the risk haplotype [5–8] as well as 3 patients carrying very uncommon haplotypes, consistent with possible hemizygosity of the same region. Four additional HSCR patients were included as positive controls: two with a de novo deletion at the centromeric region of chromosome 10 [12, 13], one with an inverted duplication at chromosome 22, and another one with a trisomy 21 (in addition to two HSCR patients already included in the sample set and presenting with Down syndrome, OMIM# 190685). The whole sample analyzed is thus constituted by 59 HSCR patients.

Besides the major *RET* gene, other candidate genes and loci were selected for the analysis based on i) linkage with HSCR, ii) association with the disease, iii) mutation in syndromic and isolated HSCR patients, iv) involvement in the transcriptional regulation of *RET*, and v) preliminary evidence, not confirmed later. Finally, other loci were included because altered in disorders presenting HSCR with a higher prevalence than the general population (Table 1).

**Table 1:** Custom array-CGH design: regions mapped and probe density

probes on the chip	candidate region	Locus	average space selected	boundaries selected (kb)	# of probes	reason for selecting^a^	references
selected	*RET*	10q11.2	300 nt	100 kb	813	linkage, mutation, association	[1]
	9q31	9q31	2.5 kb	0 kb	1824	linkage	[3]
	9p24.1	9p24.1	3.5 kb	0 kb	142	preliminary data	unpublished
	*PHOX2B*	4p13	500 nt	10 kb	49	association, transcriptional, HSCR increased prevalence	[1, 20, 21]
	*NRG1*	8p12	500 nt	10 kb	473	association	[9, 22]
	*SEMA3A/3D*	7q21.11	2.5 kb	10 kb	508	association	[10, 11, 22]
	6q25.1	6q25.1	3.5 kb	0 kb	714	preliminary data	unpublished
	21q22	21q22	50 kb	0 kb	202	HSCR increased prevalence	[1]
	3p21	3p21	3.5 kb	0 kb	1141	linkage	[4]
	19q12	19q12	3.5 kb	0 kb	1085	linkage	[4]
	*NRTN*	19p13.3	800 nt	5 kb	18	mutation	[1]
	16q23.3	16q23.3	3.5 kb	0 kb	714	linkage	[23]
	*NKX2–1*	14q13	800 nt	5 kb	17	transcriptional, mutation	[24]
	*SOX10*	22q13	800 nt	5 kb	27	transcriptional	[1]
	22q11.2	22q11.2	50 kb	0 kb	162	HSCR increased prevalence	[25]
	*ECE1*	1p36.1	800 nt	5 kb	103	mutation	[1]
	*ZEB2*	2q22.3	800 nt	0 kb	165	mutation	[1]
	*EDNRB*	13q22	800 nt	5 kb	112	linkage, mutation	[1]
	*GDNF*	5p13.1-p12	800 nt	5 kb	42	mutation	[1]
	*EDN3*	20q13.2-q13.3	800 nt	5 kb	44	mutation	[1]
genome					3130		
replicated					301 (×5)		
normalization					1262		
Agilent controls					1482		

^a^ linkage = reported in linkage with HSCR; mutation = reported as mutated in isolated HSCR; association = found associated with HSCR; transcriptional = suggested as involved in HSCR due to transcriptional evidences; preliminary data = suggested as deleted or amplified by preliminary results, later not confirmed; HSCR increased prevalence = increased prevalence of HSCR among patients with disorders caused by these genes/loci

### Aberrations detected

The selected HSCR patients and positive controls underwent custom aCGH. As reported in Additional file 1, a total of 75 calls were estimated from the raw data using the Agilent Aberration Detection method as described under Methods. Two of these calls corresponded to already known trisomies of chromosome 21, and four calls identified the alterations included as controls.

In addition, we evaluated the profiles of all the samples by visual inspection. This allowed us to add 6 calls to the list of aberrations, undetected by the software. Finally, although we did not expect any new variant in replicates, in a triplicate sample we found two aberrations that had not been detected in the array firstly investigated, but were present in both the two replicates and thus assumed as reliable, for a total of 83 aberrations detected in 64 different chromosomal locations in 44 samples (Additional file 1, Figure S1). Twenty-six of these aberrations had previously been reported on DGV; all of them were common CNVs (frequency > 5%), with the exception of the deletion at SEMA3A/3D, that is compatible with two CNVs detected by sequencing [26, 27] with an overall frequency of less than 1% (Table 2, Additional file 1: Figure S1). Four of the CNVs common in DGV were recurrent in the samples. The variants at 15q11 and 5q13, and the gain at 9p11, common on DGV, showed frequencies roughly similar to those reported for controls in the high resolution gnomAD (https://gnomad.broadinstitute.org/) and deciphering developmental disorders (DDD) (https://decipher.sanger.ac.uk/) databases [28, 29]. Losses at 9p11 and 9q31 had frequencies in between gnomAD and DDD. Interestingly, variants on 22q11.2 were all but one more frequent in our sample (Additional file 2). Moreover, three regions, found to carry anomalies in 5 samples, are compatible with CNVs reported on the Decipher database. The region 22:25672585–25,892,401 was found duplicated in two patients and deleted in a third patient, with anomalies also reported on DGV, with frequencies similar to controls in gnomAD and DDD and regarded as likely benign common CNVs. Also the deletion at 9:113025039–113,029,430 is common on DGV and likely benign, but interestingly it is reported in a patients affected by aganglionic megacolon, intellectual disability and short stature. Finally, the deletion at 1:146638075–149,224,043 is compatible with several deletions reported on decipher including the 1q21.1 recurrent microdeletion (OMIM# 612474).

**Table 2:** Variants defined as “true”

Chromosomal region (chr:start-end)	candidate region	aberrat. Type	# probes	reported on DGV	reported on decipher (# individuals)	confirmation on available replicate/s	selected for validation	sample(s) and variant # (N) ^b^	genes
1:146638075–147,824,207	genome (1q21)	loss	4	N	Y (1q21.1 recurrent microdel)	confirmed	Y	HSCR019, #21 (1)	*PRKAB2, PDIA3P, FMO5, CHD1L, BCL9, ACP6, GJA5, GJA8, GPR89B, GPR89C, PDZK1P1, LOC200030, NBPF11*
4:41746863–41,751,291	*PHOX2B*	loss	11	N	N	confirmed	Y	HSCR403, #69 (1)	*PHOX2B*
5:69288477–70,309,855	genome (5q13)	gain	3	Y (freq ≥5%)	N	not excluded	N	HSCR016, #18 (1)	*SERF1A, SMN2, NAIP, SMA4, GTF2H2B, LOC441081, DQ591060*
7:84217007–84,225,649	*SEMA3A/ SEMA3D*	loss	4	Y (freq < 1%)	N	–	Y	HSCR005, #5 (1)	(SEMA3A; SEMA3D)(SEMA3A; SEMA3D)
8:32597644–32,598,929	*NRG1*	loss	3	N	N	–	Y	HSCR045, #29 (1)	*NRG1*
9:109273643–109,275,694	9q31	loss	2	N	N	–	Y	HSCR043, #27 (1)	(TMEM38B; ZNF462)(TMEM38B; ZNF462)
9:109336464–109,348,467	9q31	gain	6	N	N	–	Y	HSCR018, #20 (1)	(TMEM38B; ZNF462)(TMEM38B; ZNF462)
9:110381888–110,401,999	9q31	gain	9	N	N	confirmed	Y	HSCR000, #1 (1)	(KLF4; ACTL7B)(KLF4; ACTL7B)
9:112078131–112,089,193	9q31	loss	5	N	N	confirmed	Y	HSCR195, #42 (1)	*EPB41L4B*
9:113025039–113,029,430	9q31	loss	2	Y (freq ≥5%)	Y (1)	–	N	HSCR415, #77 (1)	(TXN; TXNDC8)(TXN; TXNDC8)
9:43659247–43,659,512	genome (9p11)	loss (3) / gain (1)	2	Y (freq ≥5%)	N	confirmed	N	HSCR162, #38; HSCR403, #70; HSCR421, #78; HSCR426, #80 (4)	(SPATA31A6; CNTNAP3B)(SPATA31A6; CNTNAP3B)
15:20848460–22,432,687	genome (15q11)	loss (4) / gain (6)	5	Y (freq ≥5%)	N	not excluded	N	HSCR010, #13; HSCR033, #22; HSCR064, #33; HSCR160, #181; HSCR181, #39; HSCR231, #45; HSCR335, #54; HSCR382, #67; HSCR409, #74; HSCR414, #76 (10)	*NBEAP1, POTEB, CXADRP2, OR4M2, OR4N4, OR4N3P*
15:58257674–59,009,890	genome (15q21)	gain	2	N	N	–	Y	HSCR146, #35 (1)	*ALDH1A2, AQP9, LIPC, ADAM10, HSP90AB4P*
16:82200334–82,202,467	16q23.3	gain	2	N	N	–	Y	HSCR217, #43 (1)	*MPHOSPH6*
19:31954093–31,966,036	19q12	loss	5	N	N	not evaluable	Y	HSCR481, #82 (1)	(TSHZ3; THEG5)(TSHZ3; THEG5)
22:18661724–18,920,001	22q11.2	gain	7	Y (freq ≥5%)	N	not evaluable	N	HSCR58, #32 (1)	*AK302545, BC112340, DGCR6, PRODH*
22:20345868–20,499,789	22q11.2	gain	4	Y (freq ≥5%)	N	not excluded	N	HSCR335, #56 (1)	*TMEM191B, RIMBP3*
22:21494163–21,704,972^a^	22q11.2	gain	5	N	N	confirmed	N	HSCR403, #72 (1)^c^	*GGT2, POM121L7*
22:22417683–23,228,483	22q11.2	loss	15	Y (freq ≥5%)	N	–	N	HSCR014, #17 (1)	*VPREB1, ZNF280B, ZNF280A, PRAME, LOC648691, GGTLC2*
22:22781091–23,228,483	22q11.2	loss	8	Y (freq ≥5%)	N	–	N	HSCR036, #23; HSCR183, #41 (2)	*ZNF280B, ZNF280A, PRAME, LOC648691, GGTLC2*
22:23056562–23,228,483	22q11.2	loss	3	Y (freq ≥5%)	N	confirmed	N	HSCR403, #73 (1)	(GGTLC2; IGLL5)
22:25672585–25,892,401	22q11.2	loss (1) / gain (2)	5	Y (freq ≥5%)	Y (3)	not excluded	N	HSCR016, #19; HSCR228, #44; HSCR421, #79 (3)	*LRP5L, CRYBB2P1*

Variants have been defined as “true” when either reported on DGV, confirmed on a second array replicate, or validated with a different approach (see follow up in Table 4)

Genes between brackets are those flanking the CNV when no gene is affected by the aberration

^a ^not selected for validation because initially considered unlikely at the visual inspection

^b^ the code for the sample in which the CNVs has been detected is reported, together with the variant call # as indicated in the Additional file 1 and, among brackets, the number of times the CNVs has been detected in the 59 samples

^c^ this CNV was detected also in other two samples, for which neither replication nor validation through a different approach were carried out. For this reason, these are not included in the count of the true variants

In conclusion, excluding the control regions and chromosome 21 for the two Down syndrome HSCR patients, we have detected 51 novel aberrations, plus the one reported on DGV with very low frequency (Additional file 1, Figure S1). As two of these variants were recurrent in three patients each, we had a total of 48 distinct variants detected in 25 patients. Several of them looked unlikely at the visual inspection and, as a matter of fact, mainly not confirmed on the replicate, when available. Conversely, most of the variants classified as likely or possible at the visual inspection were also replicated [30] (Additional file 1).

### CNVs already reported in HSCR

Despite 41 aberrations were called at the *RET* locus in 40 samples, they were barely overlapping and rarely located on the risk haplotype. Only two of them were considered after applying the MALR> 0.30 criteria (see Methods), but no one looked as likely at visual inspection and could be confirmed at validation, proving that those CNVs were false positive. Therefore, neither deletions hypothesized based on homozigosity nor very rare haplotypes compatible with hemizygous conditions were sustained by the present data.

Most of the regions reported as duplicated or deleted in other studies [16, 17] were not included in our selected regions, neither we could detect any aberration comparable with those already reported when covered by probes in our design.

### Variant validation and parental origin

**Fig. 1:**
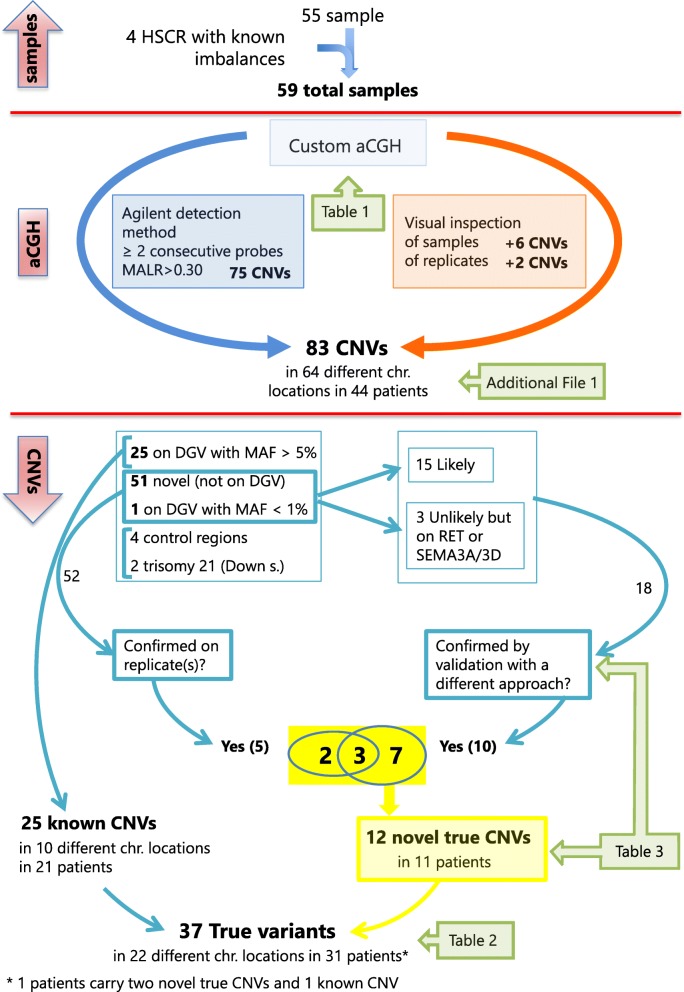
Diagram of the study design. Flowchart of the analysis performed on the complete panel of 59 patients, including the Agilent informatic method (on the left) and the visual inspection (on the right), that have led to the detection of 83 CNVs, together with the tables and files generated at each step. In particular, excluding the six already known control CNVs, the remaining 77 are further distinguished based on the DGV database (25 CNVs with a frequency higher than 5%, and thus considered true, and 52 CNVs novel or very rare on DGV) and on a visual classification. Fifteen likely true CNVs and three CNVs located on known HSCR genes have been validated by a different approach, confirming a total of 12 novel “true” CNVs in addition to the 25 already described on DGV. Numbers shown on the top of the diagram (above the red line) refer to samples, while those shown below the red line refer to CNVs (not coincident with the number of samples carrying the CNVs)

**Fig. 2:**
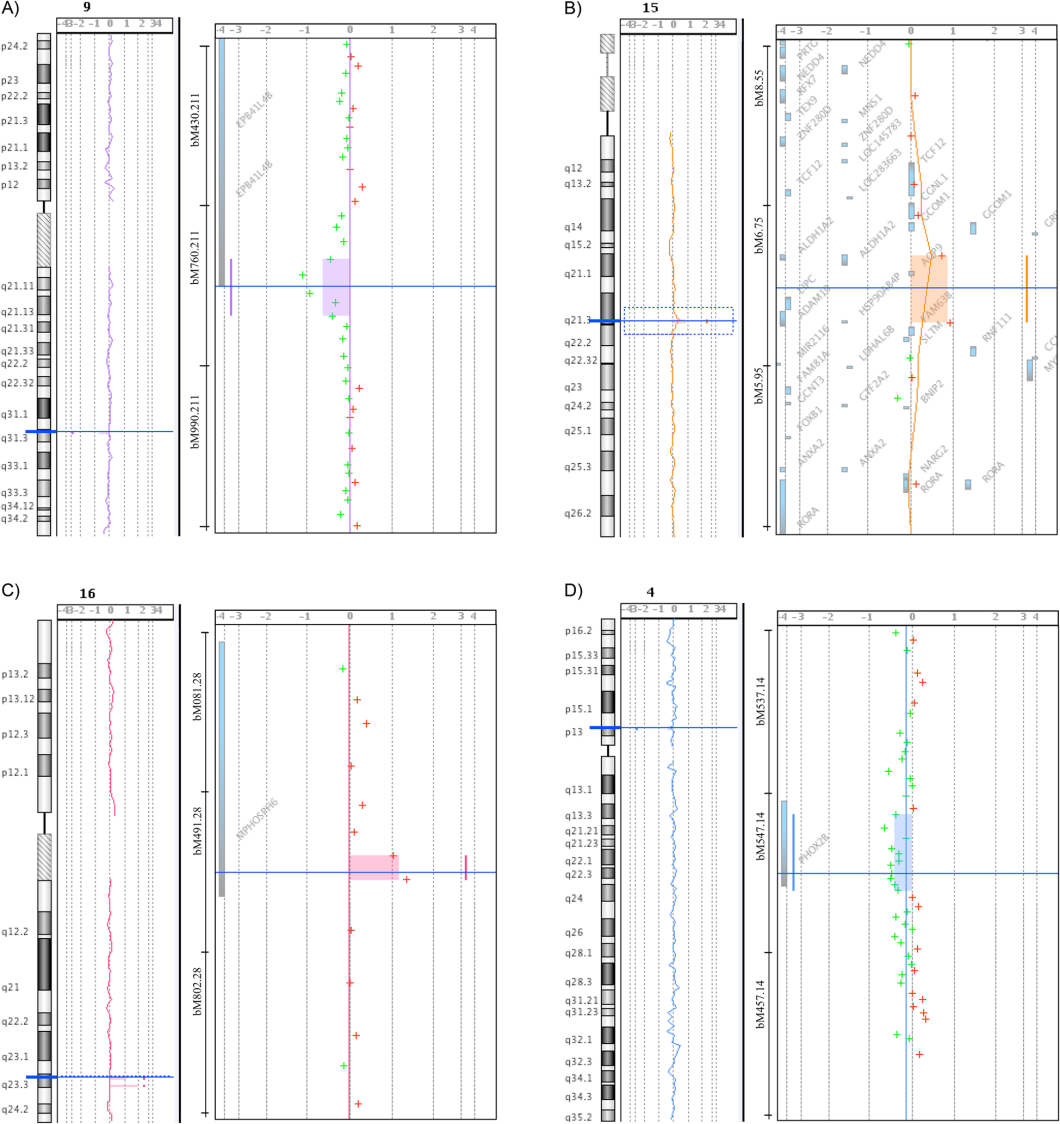
Profiles for some validated CNVs. Copy Number Variation (CNVs) detected at 9q31 (**a**), 15q21 (**b**), 16q23 (**c**) and PHOX2B (**d**) are shown. On the left of each panel there is the chromosomal view, on the middle the detailed region view with genes reported, and on the right the possible presence of CNVs

We elected to focus on gains/losses that seemed more promising at the visual inspection (classified as likely) and not reported on DGV, in addition to one deletion of the *SEMA3A/SEMA3D* region, compatible with a CNV reported on DGV but with a low frequency. We included also three aberrations unlikely at the visual inspection but particularly interesting as located in the *SEMA3A/SEMA3D* region and the *RET* locus, two master loci in HSCR development [1, 10, 11]. We have thus selected for validation 18 aberrations found in 14 patients (Table 3, Fig. 1, Fig. 2, Additional file 1).

**Table 3:** Variants selected for validation, results and corresponding samples

variant call	Sample ID	gender	Chromosomal region	Aberr. type	N. probes	size (bp)	validation method	validated	parental origin	RET mutations	RET HSCR risk haplotype	HSCR lenght	syndromic	locus	affected genes (or flanking genes if no gene maps into the region)
			(chr:start-end)												
**1**	HSCR000	F	**9:110381888–110,401,999**	**gain**	**9**	**20,111**	**qPCR**	**Y**^c^	M	interstitial deletion	unknown	TCA	no	9q31	*(KLF4; ACTL7B)*
**5**	HSCR005	M	**7:84217007–84,225,649**	**loss**	**4**	**8642**	**qPCR**	**Y**^d^	M	no	homoz.	S	no	SEMA3A/3D	*(SEMA3A; SEMA3D)*
6			10:43679892–43,680,816	loss	5	924	PCR	N	–					–	*–*
8	HSCR006	M	10:43679612–43,680,816	loss	6	1204	PCR	N	–	no	homoz.	L	no	–	*–*
**20**^b^	HSCR018	M	**9:109336464–109,348,467**	**gain**	**6**	**12,003**	**qPCR**	**Y**	M	p.P399L	wt	L	no	9q31	*(TMEM38B; ZNF462)*
**21**	HSCR019	F	**1:146638075–147,824,207**	**loss**	**4**	**2,585,968**	**qPCR**	**Y**^c^	M	no	homoz.	S	VSD + mandibular hypoplasia	1q21	*PRKAB2, PDIA3P, FMO5, CHD1L, BCL9, ACP6, GJA5, GJA8, GPR89B, GPR89C, PDZK1P1, LOC200030, NBPF11*
**27**^b^	HSCR043	M	**9:109273643–109,275,694**	**loss**	**2**	**2051**	**qPCR**	**Y**	M	p.K821Q	homoz.	S	no	9q31	*(TMEM38B; ZNF462)*
28^b^	HSCR045	M	7:84594683–84,607,065^e^	loss	6	12,382	qPCR	N	n.a.	no	unknown	L	no	–	*–*
**29**^b^			**8:32597644–32,598,929**	**loss**	**3**	**1285**	**qPCR**	**Y**						NRG1	*NRG1*
30			10:43679612–43,680,816	loss	6	1204	PCR	N						–	*–*
**35**	HSCR146	M	**15:58257674–59,009,890**	**gain**	**2**	**752,216**	**aCGH 8x60K**	**Y**	M	no	homoz.	S	no	15q21	*ALDH1A2, AQP9, LIPC, ADAM10, HSP90AB4P*
36			19:30888070–30,891,329	gain	2	3259	qPCR	N	–					–	*–*
**42**	HSCR195	M	**9:112078131–112,089,193**	**loss**	**5**	**11,062**	**qPCR**	**not conclusive** ^c^	n.a.	p.E610K	homoz.	S	no	9q31	*EPB41L4B*
**43**	HSCR217	M	**16:82200334–82,202,467**	**gain**	**2**	**2133**	**qPCR**	**Y**	de novo	p.R813L	homoz.	S	no	16q23	*MPHOSPH6*
60	HSCR349	F	10:43573685–43,574,005^e^	gain	2	320	qPCR	N	–	no	homoz.	?	no	–	*–*
61	HSCR374	F	10:43473690–43,474,033^e^	gain	4	343	qPCR	N	–	no	homoz.	L	no	–	*–*
**69**^c^	HSCR403	F	**4:41746863–41,751,291**	**loss**	**11**	**4428**	**qPCR**	**Y**	M	no	homoz.	S	no	PHOX2B	*PHOX2B*
**82**	HSCR481	F	**19:31954093–31,966,036**	**loss**	**5**	**11,943**	**qPCR**	**Y**	n.a.	no	unknown	?	Down s.	19q12	*(TSHZ3; THEG5)*
**72**^a^	HSCR403	F	**22:21494163–21,704,972**^e^	**gain**	**5**	**210,809**		**-** ^c^	**–**	no	homoz.	S	no	22q11	*GGT2, POM121L7*

CNVs reported in bold are those confirmed to be true

^a^ the CNVs reported in the last line was not actually selected for validation and not analyzed for parental origin because considered unlikely at the visual inspection, but lately found confirmed on two replicates and thus included in the present table

^b^ aberration not detected by the software call, but identified by visual inspection

^c^ confirmed on an aCGH replicate

^d^ reported on DGV (freq< 1%)

^e^ unlikely at the visual inspection

^f^ compatible with 1q21.1 recurrent microdeletion

n.a. parents not available

*VSD* VentricularSeptalDefect

Eleven of these 18 aberrations were confirmed in 11 patients (Table 3), including one variant that gave no conclusive results by validation with a different method but was confirmed on a replicate. Seven were instead not confirmed, mostly of which on *RET* and/or already considered as unlikely after visual inspection. In addition, one variant initially not selected for validation, because considered unlikely at the visual inspection, was confirmed on a replicate and resulted recurrent in two other samples that have not been further analyzed. Interestingly, some of these 12 true novel aberrations involved the NRG1, SEMA3A/SEMA3D and PHOX2B loci, three of the strongest candidates among our target regions. In particular, the short deletion detected between exon 6 and 7 of NRG1 affected a male isolated patient with L-form HSCR, the ~ 9 kb deletion in SEMA3A/3D was intergenic (between SEMA3A and SEMA3D), and was inherited from the mother by a male S-form HSCR isolated patient. We could also detect a deletion that involved almost the entire PHOX2B gene in a female isolated S-form HSCR patient, shown to be inherited by the mother. Other CNVs were found at known HSCR candidate loci 9q31 [3] (in four patients, although not overlapping between each other and covering a gene in only one patient), 16q23.3 (a short inter-exonic gain) [23], and 19q12 (not involving any known gene) [4]. In addition, a CNV recurrent in three samples has been detected at 22q11.2, a locus affected in DiGeorge and VeloCardioFacial syndromes, and in der (22) and Cat-Eye syndrome, disorders presenting HSCR with a higher prevalence than the general population [31]. Finally two CNVs, at 1q21 and 15q21, were found outside any of the HSCR candidate loci represented at high-density probes in the array (Table 3). Among these 12 losses and gains found in 11 patients, the M/F rate was 7/4, with an enrichment in females with respect to the whole sample analyzed (from 28.8 to 36.4%), seven patients had an S-form (70.0% with respect to 58.8% for the whole sample analyzed), while three were L and one unknown (Table 4). Nine were isolated cases, while one patient had Down syndrome and another one presented with cardiac and facial malformations. Finally, 5 patients carried either *RET* variants or *RET* locus deletions, with increased frequency of *RET* anomalies with respect to the original sample (45.5% vs 23.7%). Interestingly, the four patients with aberrations at 9q31 were all defective for *RET*.

**Table 4:** Summary of the anomalies found in HSCR patients subjected to the aCGH analysis

Features	patients analyzed	patients with aberration/s detected^a^	patients with “true” aberration/s^a^	patients with true aberration/s on DGV^a^	patients with true aberration/s not on DGV^a^
HSCR form					
L/TCA	21	16	12	9	3
S/ultraS	30	21	15^b^	9	7
unknown	8	5	4	3	1
gender					
M	42	29	23	16	7
F	17	13	8^b^	5	4
syndromic					
no	49	36	26^b^	18	9
yes	10	6	5	3	2
RET mutation					
no	45	31	22^b^	17	6
yes	14	11	9	4	5
5′ haplotype					
risk homo	46	31	23^b^	17	7
risk het	1	1	0	0	0
no risk	6 (3 + 3)	5	4	3	1
rare	3	3	2	1	1
unknown	3	2	2	0	2
tot patients	59	42	31^b^	21	11
tot aberrations		77 (+ 6 control regions)	37	25	12

^a^ excluded the four control regions and the region of trisomy for the two HSCR patients with associated Down syndrome. 7q21.11 del found in HSCR005 is included among those “not reported” (last column) although present on DGV database, but at a very low frequency

^b^ the sum of patients with “true” variants reported and not reported on DGV is different from the overall number of patients with “true” variants as one patient displayed two true aberrations, one on DGV and another one not on DGV

No difference in the distribution of patients’ characteristics was detected compared to the whole sample analyzed, when considering both the newly detected 77 aberrations and the “true” 37 CNVs detected. Indeed, in both cases the median size of CNVs was larger among syndromic patients than among isolated cases: 1,5 Mb vs 16.5 Kb in 9 and 68 newly detected CNVs in syndromic and isolated patients respectively, and 2.8 Mb vs 514 Kb in 5 and 32 “true” CNVs, with a borderline *p*-value (*p* = 0,0866), as already reported by others [17]. No difference was detected instead with regards to patients gender, while *RET* negative patients carried CNVs larger on average than *RET* mutated patients.

While parents were not available for three patients, and only one gain showed to be de novo, all the other seven validated CNVs resulted inherited by unaffected mothers and none by fathers (100%, exact confidence interval from 59 to 100%, *p* = 0.0078).

## Discussion

We have performed a high density custom array-CGH to search for DNA copy imbalances at selected candidate genes and loci in a total of 59 HSCR patients. Despite our interest on the *RET* gene, we could not detect any novel variant at this locus, in line with what reported by others [16–18, 25, 32]. Moreover, the *RET* locus presented with false positive calls, confirming the difficulties raised by this subcentromeric region. Also other genes, previously investigated for deletions and amplifications (*ZEB2*, *EDN3* and *GDNF*), did not show any alteration [16, 32]. At opposite, we could detect CNVs at two loci, 1q21 and 15q21 (Fig. 2), never implicated in HSCR before, by probes randomly selected to cover the whole genome at low density.

Unfortunately, given the uneven probes distribution of the present design, we could not assess the possible CNVs enrichment in HSCR candidate genes with respect to the other chromosomal regions. Nonetheless, data from controls were looked up in the high resolution databases gnomAD and DDD [28, 29] and analyzed to investigate the CNVs detected in our panel of HSCR cases. As shown in the Additional file 2, we were able to demonstrate the presence of several novel deletions/duplications in candidate genes and loci and to suggest an enrichment of common CNVs in 22q11.2 over controls. 9q31 and 9p11 losses have resulted with a frequency significantly different compared to both the control databases. However, as these latter control frequencies are very different from each other, a degree of discordance can be hypothesized between the control sets of these two databases.

Linkage of HSCR to 9q31 was shown in families with no or hypomorphic *RET* gene mutations, suggesting that these latters would require the action of other defects [3]. Novel chromosomal variants at locus 9q31 were confirmed in our dataset in 4 out of 59 patients, who also carried either heterozygous missense variants of the *RET* gene or large deletions at the *RET* locus, associations consistent with the digenic inheritance of HSCR already suggested [3, 19]. Other studies have pointed to 9q31 as a region involved in HSCR, but only a few suggestive causative genes have been identified so far. Among these, *IKBKAP* (OMIM# 603722) was found associated with HSCR in Chinese samples, especially in patients carrying *RET* coding variants [33], suggesting population specificity and implying that, in agreement with our observations, *RET* variants are found to co-occur with additional chromosomal anomalies. Interestingly, despite lack of concordance about HSCR gene(s) on 9q31, a quantitative linkage analysis carried out on genes likely involved in the enteric nervous system development identified a “master regulator” locus in 9q31 [34].

We also detected novel CNVs in *SEMA3A/3D*, *NRG1* and *PHOX2B* (Fig. 2). Class 3 Semaphorins, known to be involved in neuronal migration, proliferation, survival, and axonal guidance [35], have been demonstrated to be HSCR susceptibility factors [10, 11]. The importance of *SEMA3D* signaling in the ENS is further supported by gene expression comparison between wild-type and Ret ^k−^/^k-^ mouse gastrointestinal tracts [36]. Neuregulin 1 (*NRG1*) is essential for the development of the nervous system and the heart and its deregulation has been linked to cancer, schizophrenia and bipolar disorder (BPD) (OMIM# 181500) [37]. *NRG1* has also been identified as an additional HSCR susceptibility locus in Asian populations [9, 38]. Such an association, found initially to be below genome-wide significance in Caucasians [22, 39], has been demonstrated also in an European population [40]. Moreover, *NRG1* expression has been found to be significantly higher in HSCR than in controls tissues [41]. An interplay between *RET* and *NRG1* has been suggested [2]. We could also detect a deletion of the *PHOX2B* gene, a gene that codes for a homeodomain transcription factor involved in the development of several noradrenergic neuronal populations in the autonomous nervous system [1]. Different heterozygous mutations of *PHOX2B* are known to cause Congenital Central Hypoventilation Syndrome (CCHS) (OMIM# 209880), a rare disease characterized by impaired ventilator response to hypercapnia and hypoxia, often associated with HSCR and neuroblastomas [1]. A *PHOX2B* interstitial deletion, as well as *PHOX2B* mutations, have been reported in HSCR patients [20, 21]. Moreover, in-frame deleted and common polyA contracted alleles of the *PHOX2B* gene have shown to either abolish or reduce the transactivation activity of the mutant proteins, respectively [21, 42]. Therefore our results support *PHOX2B* loss-of-function as a rare cause of the HSCR phenotype.

We also confirmed one aberration affecting 19q12, a locus found to be in linkage with HSCR [4], and detected a de novo gain at locus 16q23.3 (Fig. 2), previously identified by a genome-wide association study in 43 Mennonite family trios [23]. The only known gene in this region is *MPHOSPH6* (M-phase PHOSPHoprotein6) (OMIM# 605500), an exosome-associated protein that is phosphorylated during mitosis [43].

In addition, we found quite large aberrations in two regions not included among the selected candidate loci. The 15q21.3 locus contains several genes, among which *ALDH1A2* (Aldehyde Dehydrogenase 1 Family, Member A2) (OMIM# 603687) is particularly interesting, coding for an enzyme that catalyzes the synthesis of retinoic acid (RA) from retinaldehyde. RA is a hormonal signaling molecule critical during embryonic development, that has already been documented as a regulator of *RET* expression in cardiac and renal development, to delay the colonization of the hindgut by *RET*-positive enteric neuroblasts, and to result in ectopic *RET* expression during embryogenesis. RA has also been proposed to maintain migratory signals and deficiency of its precursor, Vitamin A, and could therefore increase HSCR penetrance and expressivity [44]. Finally, targeted inactivation of the mouse aldh1a2 has been shown to lead to agenesis of the enteric ganglia, a condition reminiscent of human Hirschprung’s disease [44]. Another interesting gene in the same region is *ADAM10* (OMIM# 602192), a member of the ADAM family, cell surface proteins with both adhesion and protease domains, which cleaves TNF-alpha, E-cadherin, L1cam and other proteins, besides regulating Notch signaling, a process required for progenitor cell lineage specification and maintenance [45].

The deletion in 1q21.1-q21.2 spans the 1q microdeletion syndrome region, increasing the risk of delayed development, intellectual disability, physical abnormalities, and neurological and psychiatric problems. Recently, a deletion and two duplications at locus 1q21.1 have been detected in HSCR patients and reported as significantly overrepresented compared to controls, thus confirming the consistency of our result [19]. The patient carrying this maternally inherited deletion is a female with S-form HSCR without any *RET* coding variant, reported to present ventricular septal defect (VSD), mandibular hypoplasia, and low-set ears. As far as we know, this is the first case reported of 1q21 microdeletion syndrome associated with HSCR.

In our study, we have elected to focus on CNVs never reported on DGV database. Surprisingly, seven out of 8 of such novel and confirmed CNVs were inherited by the unaffected mothers, while the eighth one had a de novo occurrence. Similar observations have already been made in Type 2 Diabetes (T2D) (OMIM# 125853) and long QT syndrome (OMIM# 192500) [46, 47] and it has also been demonstrated that inherited CNVs might be pathogenic [48]. Interestingly, an increase in CNV burden in the mothers of children affected by mental retardation (MR) have been recently reported in the Chinese population, suggesting that females might be more tolerant than males to deleterious variations and that MR manifestation for females might require a higher burden of deleterious variants [49]. Consistent with our observation of an excess of inheritance from unaffected mothers, a parental mutation transmission asymmetry has already been reported at the *RET* locus [50]. Apparently, this bias in the transmission of *RET* single base mutations was not due to different expression of the disease depending on the gender of the transmitting parent, but rather to differential reproductive rate between male and female carriers, with mothers carrying a severe mutation that would be more likely than fathers to reproduce and transmit [50]. Considering that HSCR penetrance is less reduced in male than females, with recurrent risk for male sibs higher when the HSCR patient is female [1, 5], the differential paternal and maternal fitness and the supposed greater mutation burden toleration in females seem to be the two faces of the same coin. Therefore, asymptomatic females carrying these HSCR structural variants would be more likely to transmit the causative CNV alleles, as already demonstrated in other diseases, especially in the presence of additional alterations.

## Conclusions

Three fundamental genes already involved in HSCR pathogenesis, namely *SEMA3A/3D*, *NRG1* and *PHOX2B*, have been shown in our study to play a role also through the presence of CNVs, thus suggesting their haploinsufficiency is responsible of damaging effects. Indeed, to our knowledge the presence of structural variants in HSCR patients has never or rarely been reported for *NRG1*, *SEMA3A/3D*, and *PHOX2B*. Conversely *RET* involvement in HSCR does not seem to rely on the presence of CNVs but, interestingly, several gains and losses did co-occur with another *RET* defect in our sample, thus sustaining the hypothesis that more than one predisposing event is necessary for HSCR to develop. Our results, not surprisingly for a complex genetic disease like HSCR, support a role of candidate genes in transcription and expression regulation and in ENS development, confirming the known genetic heterogeneity and showing the possible involvement of new loci. Finally, all the CNVs shown to be inherited in our samples were of maternal origin, including the four novel CNVs detected on 9q31 affecting patients who were also carrying variants of the *RET* proto-oncogene.

## Methods

### HSCR patients and microarray design

A total of 55 Italian sporadic HSCR patients were retrospectively included in the study, as described under the “Results” section.

We performed high-resolution oligonucleotide array-CGH analyses on 20 candidate genes/loci known to be involved in HSCR, using a customized 8x15K array (Agilent Technologies, Santa Clara, CA, USA), in accordance with manufacturers’ instructions, whose details are reported in Table 1. Additional probes were also distributed along the whole genome, including those useful for sample replication and normalization. Genomic positions are based on the Human Genome GRCh37 (hg19) assembly (http://genome-euro.ucsc.edu).

### Data analysis

To assess genomic imbalances, we applied the Aberration Detection Methods ADM-2 with a threshold of 6, as recommended by Agilent. We also applied the centralization and the GC correction algorithms and considered as aberrant only those regions with a minimum of 2 consecutive probes and exceeding a Mean Absolute Log2 Ratio of 0.30 (referred as MALR> 0.30). In addition, sample profiles were evaluated at the whole genome level by visual inspection. Such a manual search was performed paying attention to log_2_ ratios values above 0.5 and below − 1.0 and taking into consideration not only the overall profile of the single individual sample (and its quality) but also the profiles of the entire cohort. The above parameters were kept, with the exception of the MALR> 0.30 filter, and observations were confirmed by a second operator. Based on such inspection, we classified the CNVs detected as “likely”, “possible”, or “unlikely”. Loci with nearby gain or loss intervals and an intervening region of more than 2 probes were considered as separate CNVs, as well as those differing for 2 probes with opposite log_2_ ratios (log_2_ratio < − 0.3 for gain and > 0.3 for deletions). The quality of the experiments was evaluated on the basis of the QC metrics generated by the Genomic Workbench 5.0.14 software (Agilent Technologies), such as the DLRSpread (derivative log ratio spread), a measure of the log ratio noise for each sample. DLRSs and the other sample metrics are detailed elsewhere [30].

Ten samples with bad profiles (DRLS ≥0.3) together with other 16 arbitrarily selected samples, were replicated at least once on another array, for a total of 26 samples with at least one replicate. The successive variants search was performed in the replicates of overall better quality.

Aberrations were compared with CNVs observed in the normal population, as reported in the Database of Genomic Variants (DGV, http://dgv.tcag.ca/dgv/app/home), and with the CNVs reported in the DECIPHER database of phenotypes, v8.7 release (https://decipher.sanger.ac.uk/). Data comparing is challenging as exact boundaries of the aberrations are not known, but only assumed to be between the last “normal” probe (outer) and the first “aberrant” probe (inner), depending on the mean coverage. However, we considered aberrations as consistent with those already reported if they showed an overlap ≥80%, did not differ for more than two probes with compatible log_2_ ratios (that is ≥ |0.3|), and were of the same kind (gain or loss).

To get further insight into the aberrations thus identified, we also compared their frequencies in our sample with frequencies of corresponding CNVs publically available at a resolution similar to that of our design, considering comparable those CNVs with identical boundaries (Additional file 2). To this end we used the control data from the European population in the GnomAD website (https://gnomad.broadinstitute.org/) and from the DDD database, browsed through the UCSC genome browser (https://genome-euro.ucsc.edu/index.html), and assessed statistical differences through the Fisher’s test or the Chisquare test with Yate’s correction for continuity when more feasible.

### Validation

Results obtained with the custom aCGH, together with the concordance degree among the replicates on the same design array, showed that the replication rate was not very high, and that the visual inspection outperformed the mere software call [30]. However, a high false positive rate is not surprising as a few studies have shown a not infrequent presence of false positive and false negative results from aCGH [51–54]. As a matter of the fact, Conrad et al. (2010), using quantitative PCR (qPCR) for initial validation of aCGH experiments on 450 HapMap samples, suggested a preliminary false-discovery rate of ~ 20%, then assessed to 15% when comparing the results with another CNV-typing array [55].

For this reason, the most promising regions, based on rare presence/absence on DGV and visual inspection, were selected to undergo validation with different approaches. Parents were also investigated, when available, to verify whether the aberrations were inherited or de novo. When the deleted region was sufficiently small, the DNA was amplified with PCR and checked for anomalous bands by electrophoresis. For the majority of the aberrations, validation was carried out applying quantitative PCR. Primer pairs were designed with Primer-blast (http://www.ncbi.nlm.nih.gov/tools/primer-blast/) according to stringent parameters to ensure successful assay. qPCR analysis was performed with the LightCycler 480 Instrument and relative software using the SYBR Green I Master following manufacturer’s recommendations (Roche, Manheim, Germany). In case of inconclusive results, we spotted the sample on a CGH 8x60K array (Agilent).

The probability that 7 out of 7 inherited CNVs were transmitted by mothers was checked by the binomial test.

Finally, we defined variant as “true” when either reported on DGV, confirmed on a second array replicate, or validated with a different approach as described above.

## Supplementary information

**Additional file 1.** “Variants detected by software and visual inspection, according to selection criteria”. Whole raw data obtained by applying the a-CGH approach.

**Additional file 2.** “Frequencies of CNVs detected in our sample compared to those for controls from gnomAd and DDD”. Comparison of all the aberrations detected by aCGH in our study and the frequencies reported for controls in two databases.

## Data Availability

All CNV data generated and analyzed during this study are included in this published article and in particular in the Additional file 1.
